# Mean Platelet Volume Enhances the Diagnostic Specificity of PSA for Prostate Cancer

**DOI:** 10.3389/fsurg.2022.845288

**Published:** 2022-04-12

**Authors:** Wei Song, Ning Ding, Xiulin Zhang, Jiaxin Liu, Yuzhen Wang, Jieke Yan, Shuangde Liu

**Affiliations:** ^1^School of Basic Medical Sciences, Cheeloo College of Medicine, Shandong University, Jinan, China; ^2^Department of Urology, Shandong Provincial Hospital Affiliated to Shandong First Medical University, Jinan, China; ^3^Department of Urology, The Second Hospital, Cheeloo College of Medicine, Shandong University, Jinan, China; ^4^Department of Kidney Transplantation, The Second Hospital, Cheeloo College of Medicine, Shandong University, Jinan, China; ^5^Clinical Department, Jinan Nursing Vocational College, Jinan, China

**Keywords:** prostate cancer, platelet distribution width, prostate specific antigen, receiver operating characteristic, platelet distribution width (PDW)

## Abstract

Mean platelet volume (MPV) is an indicator of platelet activation and has been proposed as a diagnostic marker for several kinds of cancers. We investigated the value of MPV as a diagnostic marker for prostate cancer (PCa) and examined whether MPV in combination with prostate-specific antigen (PSA) could increase the sensitivity or specificity of PSA for PCa diagnosis. For this study, 107 pathologically confirmed PCa and 177 non-PCa patients who underwent prostate biopsy were retrospectively studied. Clinical data and pre-biopsy hematological parameters were collected. The above parameters were compared between PCa and non-PCa patients. The correlation between MPV and clinical characteristics was analyzed. Receiver operating characteristic (ROC) analysis was used to explore the diagnostic value of MPV for PCa. Among all parameters analyzed, the difference was only found in MPV, platelet distribution width (PDW), and PSA between PCa and non-PCa patients. MPV was significantly decreased and PDW increased in PCa than that of non-PCa among men. ROC analysis identified MPV ≤ 9.05 fl as a cut-off value for potential PCa with area under the ROC curve (AUC) = 0.783, 95% CI = 0.733–0.833, sensitivity = 0.746, and specificity = 0.708. AUC and the sensitivity of MPV were comparable with total PSA (TPSA) or free PSA (FPSA). However, the specificity of MPV was larger than that of TPSA (0.461) or FPSA (0.561). Furthermore, MPV combined with TPSA or FPSA further enhanced the specificity of TPSA (0.844) or FPSA (0.927), but PDW did not. These findings suggested that MPV could have a predictive value for the diagnosis of PCa. MPV in combination with TPSA or FPSA could enhance the specificity of PSA and may reduce the rate of unnecessary biopsy for patients with high levels of PSA.

## Introduction

Prostate cancer (PCa) is the most diagnosed cancer and the second leading cause of cancer-related death for men in the United States (1, 2). The incidence of PCa is increasing in most Asian countries including China (3). Prostate-specific antigen (PSA) is the preoperative diagnostic of this disease and has been widely employed in predicting the pathological features of PCa. Although PSA has improved the early detection rate of PCa, the low sensitivity and specificity of PSA are often associated with over-diagnosis and lack of tumor specificity (4). Also due to the low specificity of PSA, unnecessary biopsy rates are high (5). Other novel cost-effective and convenient hematological biomarkers with higher predictive sensitivity and specificity need to be explored (6, 7).

Recent investigations showed that platelets play critical roles in mediating the growth, dissemination, and angiogenesis of cancer cells (8). Activated platelets are associated with cancer progression and metastasis (9). Mean platelet volume (MPV) and platelet distribution width (PDW) are markers of activated platelets and are associated with gastric cancer, ovarian cancer, lung cancer, colon cancer, and breast cancer (10–14). MPV and PDW have been shown to have diagnostic values for these cancers.

Several studies also revealed the association of MPV and PDW with prostate cancer (15–17). It is reported that decreased MPV and increased PDW were found in patients with PCa compared to patients with benign prostatic hyperplasia (BPH) who underwent surgery (16). They concluded that MPV and PDW combined with PSA could differentiate PCa from BPH patients. In clinical practice, a biopsy was usually conducted on suspected patients with PCa with a PSA > 4 ng/ml, and unnecessary biopsies were not in the minority. Therefore, the current study aimed to determine the diagnostic value of MPV and PDW in a population of patients who underwent biopsy (PSA > 4 ng/ml). This is to mainly see whether MPV and PDW in combination with PSA could increase the sensitivity or specificity of PSA for PCa identification, and to finally avoid unnecessary biopsy.

## Materials and Methods

### Study Subjects and Inclusive or Exclusive Criteria

This study was approved by the Ethics Committee of the Second Hospital of Shandong University. We retrospectively investigated 284 patients admitted to the Second Hospital of Shandong University between July 2015 and 2020 for prostate biopsy because of high PSA levels (>4 ng/ml). Among them, 107 had pathological confirmed PCa, and 177 had pathological non-PCa who was proved to be benign prostatic hyperplasia (*n* = 160) or normal (*n* = 10) by histology. For the 160 patients with BPH, their lower urinary tract symptom (LUTS) is not severe enough for surgical treatment, hence included in this study. Individuals with long-term administration of drugs that could affect blood examination (anticoagulant, statins, and acetylic salicylic acid) were excluded. Patients with hematological disorders, hypertension, or diabetes mellitus were also excluded.

### Data Collection

For each patient, clinical, laboratory, and pathological data were collected. Clinical data included general information (age, weight, and race), medical history, and physical examination. Laboratory data included total PSA (TPSA), free PSA (FPSA), ratio of TPSA to FPSA, and complete blood count analysis, which examined the number of platelets, white blood cell (WBC), neutrophil, lymphocyte, monocyte, eosinophils, basophils, platelet, MPV, and PDW. For pathologically confirmed patients with PCa, the information of Gleason scores and tumor, nodes, and metastases (TNM) stage were also recorded. Laboratory examinations were done in the Second Hospital of Shandong University with a biochemical analyzer which is hisemikon blood cell instrument XN9000 (Roche, Basel, Switzerland). The serum PSA was measured using an automatic electrochemistry luminescence immunoassay system (CLEIA) (Roche, Basel, Switzerland) (18). Complete blood count analysis was performed immediately within half an hour after collection of blood samples into EDTA tube after venipuncture to prevent platelet activation *in vitro*.

### Statistical Analysis

Data were calculated as mean ± *SD*. The basic characteristics of PCa and non-PCa patients were compared by *t*-test. The correlation between MPV and the clinical characteristics was evaluated using the Spearman correlation test. Receiver operating characteristic (ROC) analysis was used to explore the diagnostic values of MPV, PSA, and their combination. All analysis was conducted by SPSS (version 18, SPSS Inc., Chicago, IL, USA). The *P*-value < 0.05 was regarded as a statistical difference.

## Results

### Comparison of Parameters Between PCa and Non-PCa Patients

The clinical and laboratory data of PCa and non-PCa patients are shown in Table 1. The MPV was significantly less in PCa (9.06 ± 1.07%) than non-PCa among men (9.41 ± 1.11 fl, *P* = 0.001). On the other hand, PDW was significantly increased in PCa (15.43 ± 1.87%) than non-PCa among men (14.79 ± 2.32 fl, *P* = 0.01). The platelet count among men with PCa (232.22 ± 67.59 × 10^9^/L) tended to be more than that of the non-PCa study group (217.97 ± 69.81 × 10^9^/L), but there was no statistical difference (*P* = 0.09). Both TPSA and FPSA were larger in PCa than non-PCa among men (both *P* < 0.05; Table 1). There is no statistical difference in age between PCa and non-PCa among men. No difference was found for the count of WBC, neutrophil, lymphocyte, monocyte, eosinophils, and basophils between PCa and non-PCa among men (*P* > 0.05; Table 1).

**Table 1:** The basic information of prostate cancer (PCa) and non-PCa in men.

**Basic information**	**PCa men**		**Non-PCa men**		***t***	***P***
	***n***	$\bar{X}\pm \text{s}$	***n***	$\bar{X}\pm \text{s}$		
Age	107	68.25 ± 7.09	177	68.97 ± 7.75	3.63	0.58
TPSA	107	27.41 ± 3.34	177	15.26 ± 3.27	5.06	0.001
FPSA	107	3.13 ± 0.55	177	2.09 ± 1.03	2.78	0.006
FPSA/TPSA	107	0.12 ± 0.07	177	0.15 ± 0.08	−3.62	0.001
WBC	107	6.83 ± 2.32	177	6.38 ± 2.55	1.48	0.14
Neutrophil	107	4.51 ± 2.19	177	4.12 ± 2.27	1.39	0.17
Lymphocyte	107	1.69 ± 0.54	177	1.64 ± 0.59	0.77	0.44
Monocyte	107	0.47 ± 0.19	177	0.46 ± 0.23	0.35	0.73
Platelet	107	232.22 ± 67.59	177	217.97 ± 69.81	1.69	0.09
Eosnophils	107	0.14 ± 0.03	177	0.14 ± 0.02	0.29	0.77
Basophils	107	0.03 ± 0.01	177	0.03 ± 0.01	1.09	0.27
MPV	107	9.06 ± 1.07	177	9.41 ± 1.11	−2.63	0.009
PDW	107	15.43 ± 1.87	177	14.79 ± 2.32	2.45	0.015

*Comparisons were made by t-test. There is a statistical difference in free prostate-specific antigen (FPSA), total PSA (TPSA), FPSA/TPSA, mean platelet volume (MPV), and platelet distribution width (PDW)*.

### Association of MPV or PDW With Clinicopathologic Characteristics of PCa Patients

The relationships between MPV or PDW level and clinicopathologic characteristics in PCa patients are listed in Tables 2, 3. We did not find a significant correlation between MPV or PDW level and the TNM stage (*p* > 0.05; Table 2). Also, no significant correlation was found between MPV or PDW level and age, PSA level, or Gleason score in patients with PCa (*p* > 0.05; Table 3).

**Table 2:** Comparisons between mean platelet volume (MPV) and tumor, nodes, and metastases (TNM) in men with PCa.

**Variables**		**MPV (fL)**		**PDW (%)**	
	***n***	$\bar{X}\pm \text{s}$	***P***	$\bar{X}\pm \text{s}$	***P***
Clinical T stage			0.283		0.413
T_1_-T_2_b	74	9.11 ± 1.21		15.55 ± 2.07	
≥T_2_C	33	8.97 ± 0.94		15.16 ± 1.0.77	
Lymph node status			0.394		0.252
Negative	93	9.09 ± 0.74		15.46 ± 1.97	
Positive	14	8.91 ± 0.79		15.23 ± 1.69	
Distant metastasis			0.327		0.387
No	93	9.07 ± 0.54		15.45 ± 1.78	
Yes	14	9.03 ± 0.69		15.29 ± 1.75	

*There is no statistical difference between MPV or PDW and clinical T stage, lymph node, and distant metastasis in men with PCa (P >0.05)*.

**Table 3:** Correlation analysis between MPV or PDW and age, TPSA, FPSA, FPSA/TPSA and Gleason score in PCa patients.

**Correlation analysis**	**MPV**		**PDW**	
	**r(s)**	**P**	**r(s)**	**P**
Age	0.019	0.848	0.114	0.245
TPSA	−0.041	0.675	−0.089	0.366
FPSA	0.004	0.971	−0.051	0.606
FPSA/TPSA	0.022	0.829	0.054	0.591
Gleason score	−0.091	0.352	0.199	0.140

*No association was found between MPV or PDW and these parameters (P > 0.05)*.

### Diagnostic Values of MPV or PDW for PCa

To explore the ability of MPV or PDW to diagnose PCa, ROC analysis was made. ROC analysis (Table 4) identified MPV ≤ 9.05 % as a cut-off value for potential PCa with area under the ROC curve (AUC) = 0.783, 95% CI: 0.733–0.833, sensitivity = 0.746, and specificity = 0.708. PDW ≥ 9.20% served as a cut-off value for potential PCa with AUC = 0.796, 95% CI: 0.745–0.847, sensitivity = 0.758, and specificity = 0.72.

**Table 4:** Diagnostic value of MPV, PDW, TPSA, and FPSA biomarkers for PCa.

**Variables**	**AUC**	**Cut off**	**Sensitivity**	**Specificity**	**95% CI**	
					**Upper limit**	**Lower limit**
MPV	0.783	9.050	0.746	0.708	0.733	0.833
PDW	0.796	9.200	0.758	0.720	0.745	0.847
TPSA	0.771	10.210	0.816	0.461	0.721	0.822
FPSA	0.704	1.700	0.682	0.561	0.647	0.701
TPSA/ FPSA	0.687	0.976	0.666	0.547	0.631	0.684
MPV^*^TPSA	0.800	−0.148	0.644	0.844	0.753	0.847
MPV^*^FPSA	0.731	−0.843	0.472	0.927	0.677	0.785
MPV^*^TPSA^*^FPSA	0.799	−0.576	0.871	0.564	0.751	0.846
PDW^*^TPSA	0.813	−0.586	0.886	0.574	0.764	0.861
PDW ^*^FPSA	0.743	−0.536	0.810	0.524	0.698	0.787
PDW ^*^TPSA^*^FPSA	0.812	−0.585	0.885	0.573	0.763	0.860

Combinations did not increase the AUC, but significantly enhanced the specificity of PSA (MPV^*^TPSA, MPV^*^FPSA).

Then, we compared the diagnostic value of MPV or PDW with TPSA or FPSA (Table 4). Consistent with previous studies (18), either TPSA or FPSA has a low specificity for PCa diagnosis (Table 4). The diagnostic ability (AUC) and the sensitivity of MPV or PDW were comparable with TPSA or FPSA, but the specificity of MPV (0.708) or PDW (0.72) was much higher than TPSA (0.461) or FPSA (0.561) (Table 4).

The combination of several biomarkers might lead to enhanced sensitivities and specificities. Next, we examined the ROCs of MPV or PDW in combination with TPSA (MPV^*^TPSA, PDW^*^TPSA) or FPSA (MPV^*^FPSA, PDW^*^FPSA) (Table 4) or the three combinations (MPV^*^TPSA^*^ FPSA, PDW^*^TPSA^*^ FPSA). Compared with each alone, the AUC did not increase among MPV^*^TPSA, PDW^*^TPSA, MPV^*^FPSA, PDW^*^FPSA, or the three combinations. However, compared to each alone, the three parameters combination significantly increased the sensitivity but reduced the specificity (Table 4). To note, the specificity was much enhanced by MPV^*^FPSA or MPV^*^TPSA than each alone, and not by PDW. Most remarkably, the specificity was increased from 0.461 (TPSA alone) and 0.561 (FPSA alone) to 0.844 (MPV^*^TPSA) and 0.927 (MPV^*^FPSA).

## Discussion

The level of preoperative MPV has been found to be associated with many kinds of cancers. Our study showed that MPV in patients with PCa was significantly less than that of non-PCa among men. ROC analysis showed that MPV has the predictive value for PCa occurrence. No relationship was found between MPV level and TNM stage, Gleason score, and age, which indicated that MPV could be used in the diagnosis of patients with PCa independently of their cancer stage. ROC analysis further showed that MPV alone or in combination with PSA could enhance the specificity of PSA for PCa diagnosis. Thus, MPV should be considered as a valuable new biomarker for the correct identification of PCa among patients diagnosed with this disease.

Mean platelet volume is correlated with platelet function and may be a more sensitive index than platelet number as a marker of clinical interest in various disorders (19). Our study showed that the MPV of PCa in men was statistically less than those without PCa. This finding is consistent with the other two studies that MPV is significantly reduced in PCa than BPH (16) or patients with non-symptomatic prostatitis (20). The underlying mechanism for the decreased MPV in patients with PCa is not clear for us. It has been suggested that the level of MPV depends on the intensity of the systemic inflammation (21, 22). Mild system inflammation leads to platelet activation and enhanced release of larger platelets, therefore elevating MPV level. While severe system inflammation was associated with low levels of MPV due to increased release rate of small size platelets and selective consumption of large amounts of highly reactive large-sized platelets (21). The relationship between systemic inflammatory response and prostate cancer had been reported (23, 24). However, the role of MPV in cancer development is complicated (21), so it is unreasonable to conclude that a low level of MPV in patients with PCa may indicate severe system inflammation. Thrombosis is another factor that can decrease MPV and might be closely associated with poor survival in cancer patients (21, 25). However, no studies reported the relationship between thrombosis and PCa. Furthermore, no association was found in our study between MPV and PSA level, TNM stage, and Gleason score of PCa patients. Thus, the mechanisms and their role of decreased MPV in PCa progression require further study.

We did not find the correlation of MPV or PDW level with PSA level in PCa patients. A negative correlation of MPV with PSA was identified in one study (20) showing a higher level of MPV in non-symptomatic patients than those with BPH or PCa. This inconsistency may arise from the different patient populations included in our and their study. The correlation was analyzed only in patients with PCa in our study, whereas patients with BPH and non-symptom prostatitis were all included in the aforementioned study.

Prostate-specific antigen was extensively used as a marker for screening and diagnosis of PCa, however, PSA may be elevated because of several reasons, including prostatitis or BPH in addition to prostate cancer. Thus, as the diagnostic marker of PCa, the sensitivity and specificity of PSA are low, and over-diagnosis and unnecessary biopsy often occur (18). Consistent with one previous study [22], MPV could differentiate PCa from BPH, our study also found MPV has a value for differentiating patients with PCa from non-PCa ones in a population of patients with PSA > 4 ng/ml and underwent biopsy. Most importantly, our finding showed that MPV alone or in combination with TPSA or FPSA could enhance the specificity of PSA. Thus, pre-biopsy MPV would help to prevent individuals from getting unnecessary biopsies. Men with a low MPV level and an increase of serum PSA should strongly be considered for biopsy, whereas men with a high MPV level and an increase of PSA may not need a biopsy.

## Limitations

Our study is retrospectively conducted, and the accuracy of our results requires validation by future prospective studies. The second limitation is that the subject population from a single medical center is relatively small, so a larger population of patients from multi-medical centers is needed. Another limitation of our study is that all our patients are hospitalized patients for prostate biopsy who have a PSA level larger than 4 ng/ml. Patients with PSA less than 4 ng/ml were missed for analysis, thus, MPV may not be used as a screening test for PCa.

In summary, the present study revealed that MPV in combination with PSA could increase the specificity for differentiating patients with PCa from non-Pca ones. Because the measurement of MPV is easy, routine, and inexpensive, clinicians should check MPV in suspected patients with PCa. Men with a low MPV level and an increase of serum PSA should strongly be considered for biopsy. However, our results need to be validated with prospective and randomized investigations.

## Data Availability Statement

The raw data supporting the conclusions of this article will be made available by the authors, without undue reservation.

## Ethics Statement

The studies involving human participants were reviewed and approved by Second Hospital of Shandong University. The patients/participants provided their written informed consent to participate in this study.

## Author Contributions

All authors listed have made a substantial, direct, and intellectual contribution to the work and approved it for publication.

## Funding

This work was supported by the National Nature Science Foundation of China (No. 82070783), and the National Science Fund of Shandong Province (ZR2021MH283).

## Conflict of Interest

The authors declare that the research was conducted in the absence of any commercial or financial relationships that could be construed as a potential conflict of interest.

## Publisher's Note

All claims expressed in this article are solely those of the authors and do not necessarily represent those of their affiliated organizations, or those of the publisher, the editors and the reviewers. Any product that may be evaluated in this article, or claim that may be made by its manufacturer, is not guaranteed or endorsed by the publisher.
